# The underlying mechanism of partial anterior cruciate ligament injuries to the meniscus degeneration of knee joint in rabbit models

**DOI:** 10.1186/s13018-020-01954-6

**Published:** 2020-09-18

**Authors:** Dalin Wang, Zhe Wang, Mingcheng Li, Songbao Xu

**Affiliations:** 1 Department of Orthopaedic Surgery, Associated Hospital, Beihua University, Jilin, 132013 Jilin People’s Republic of China; 2 Department of Clinical Diagnosis, School of Laboratory Medicine, Beihua University, No.3999, East Road of Binjiang, Jilin, 132013 Jilin People’s Republic of China; 3 Department of Orthopaedic Surgery, the First Hospital of National Petroleum Industry Co. Ltd, Jilin, 132015 People’s Republic of China

**Keywords:** Anterior cruciate ligament, Anteromedial bundle, Posterolateral bundle, Meniscus degeneration

## Abstract

**Background:**

The diagnosis, treatment, and efficacy evaluation of anterior cruciate ligament (ACL) partial rupture remains controversial. This research aims to investigate the underlying mechanism of partial ACL injuries to the meniscus degeneration in the rabbit knee.

**Methods:**

Sixty New Zealand white rabbits were randomly divided into three groups including an experimental group, a sham group (*n* = 6), and a blank control group (*n* = 6). The experimental group is composed of an anteromedial bundle (AMB) rupture group (*n* = 24) and a posterolateral bundle (PLB) rupture group (*n* = 24). Rabbits in the experimental group were subjected to right hind limbs knee surgery to induce ACL part injury under the arthroscopy. Finally, eight rabbits including 6 in the model group and 2 in the control group were sampled randomly on the 2nd, 4th, and 8th weeks respectively. We observed the typical form of the meniscus through HE staining. Expressions of inflammatory factors including interleukin-1β (IL-1β) and IL-17 in the knee joint fluid were determined by means of an ELISA. Analysis of the mRNA expressions of matrix metalloproteinases-13(MMP-13) was performed to evaluate the inflammatory mediators in the pathogenesis of the meniscus.

**Results:**

HE staining results showed that the surface was rough and the tissues were loose displaying collagen fibers of varying thickness. Both IL-1β and IL-17 in the synovial fluid and the positive rate of MMP-13 in addition to MMP-13 mRNA showed a demonstrable increase treads from the 2nd to the 8th week. The significant difference was found (*P* < 0.05) compared to the control group.

**Conclusion:**

We conclude that the elevated levels of IL-1β and IL-17, along with increased MMP13 expression, resulted in meniscus degradation in the rabbit knee joint model with partial ACL injury.

## Background

The anterior cruciate ligament (ACL) is prone to be the most ruptured ligament in the knee sporting injuries. With the increasing numbers of an aging population sport and the increase in traffic accidents, ACL rupture has become an extremely common injury, which accounts for about half of all knee ligament injuries in China [1]. Clinically, the severity of ACL injury is divided into three types: mild, moderate, and severe rupture. In the cases of moderate and severe injuries, ACL reconstruction surgery is recommended. Injury to one-quarter of a single bundle of the ACL are commonly referred to as a mild injury or termed partial ACL injury [2]. ACL partial tears, which were damaged to one of the two ACL fiber bundles, were difficult to diagnose without showing accurate joint biomechanics with MRI examination; moreover, injury assessment of its functional retention with direct arthroscopic examination also was difficult owning to its diverse physical characteristics [3, 4]. Subsequent to an ACL partial or complete injury, the relationship between passive stability and the functional stability of the knee joint is sometimes ambiguous [5]. Moreover, the diagnosis, treatment, and efficacy evaluation of the partial ACL injury remain challenging with conflicting reports in the literature [6]. When the ACL part tear develops, poor knee stability results in a greatly increased probability of joint injury. Therefore, treatment of partial ACL is related to aspects of the patient’s knee joint, such as a meniscus, articular cartilage, and other structures of acute or long-term damage.

Lohmander et al. first evaluated the effects of different levels of cytokines in human knee joint fluid after injury to the cruciate ligament or meniscus in 1994 [7]. For the past two decades, numerous studies have reported elevated levels of inflammatory cytokines within the joint environment were associated with knee trauma in addition to knee osteoarthritis (OA) of progression [8]. Additionally, matrix metalloproteinases (MMP_S_) are a class of enzymes widely present in the connective tissue of the articular cartilage extracellular matrix (ECM). Physiological and pathological degradation of ECM plays an important role in the protease super-family, which can be divided into various sub-types according to dissimilar substrates. MMP-13 was selected as a bio-marker of ligament damage as it belongs to the collagenase sub-type of MMPs, which can directly degrade type 2 collagen in the cartilage matrix and reflect the metabolic changes in cartilage matrix [9, 10].

This study was designed to establish a model of discrete partial ACL bundle injuries in the knee joint of rabbits and to observe whether the differences in the morphology and histology of the meniscus are impacted by partial ACL different functional bundle injuries on the knee meniscus. In addition, we aimed to determine the significance of inflammatory factors in the articular fluid of meniscus degeneration. We also presented a predictive factor of partial ACL injury evolution into meniscus degeneration based on the pre-clinical examination in animal models.

## Methods

### Animals and groups

This experiment was approved by the Animal Experiments Ethics Committee of the Associated Hospital of Beihua University (Ethical approval number: Protocol Number 2017-08-16). Animal care was in accordance with the Animal Research: Reporting in Vivo Experiments guidelines. The study was conducted on 60 skeletally mature New Zealand rabbits (23 weeks old) weighing between 2.5 and 3.0 kg. All healthy SPF-grade male animals were obtained from the Experimental Animal Breeding and Research Centre, Bethune Medical College Animal Experimental Center, Jilin University (No.: SCXK-2018-0006). Based on previous experience, the animals were randomly assigned into three groups using a random contrast method and random number tables including an experimental group, a sham group (*n* = 6), and a blank control group (*n* = 6). The experimental group is composed of an anteromedial bundle (AMB) rupture group (*n* = 24) and a posterolateral bundle (PLB) rupture group (*n* = 24).

### Plan-operative procedure

Rabbits in the model group were subjected to right hind limbs knee surgery to induce ACL part injury under the arthroscopy (Andover, MA, USA; 72200616) as previously described [2, 10]. In a brief, the rabbits were fixed in a supine position on the operation table with the 4 cm × 4 cm surgical area shaved. Anesthesia was administered intramuscularly at dosages of ketamine (100 mg/kg), disinfected with complexed iodine, and draped in a sterile surgical towel. An adequate opening was built with 0.5-cm-long incisions through the anterior medial and lateral approach into the joint cavity. The operator explored the intact structures of articular cartilage and meniscus. While completely bleeding was stopped and fixed the knee at 0°, the ACL was fully exposed. In the AMB rupture group, a special blunt hook was used to hold the tension fibers in the middle and lower in 1/2 of the ACL and then cut off the bundle. In the PLB rupture group, firstly, flex the knee to 90; application of the special hook is to hold the loose fiber bundle in the middle and lower 1/2 of ACL; afterwards, cut off the fiber bundle while the knee joint is straightened. In the sham operation group, the same procedure as the medial incision of the knee into the joint cavity was exposed without cutting off the ACL. The anterior drawer test confirmed that the model of ACL rupture had been successfully created while the incision was then sutured with 3/0 absorbable sutures. Four days after surgery, all postoperative rabbits were intramuscularly injected with 400,000 U penicillin to prevent infection. In the blank group, the rabbit legs did not make any interventions with normal feeding. All rabbits were housed with sub-cage feeding. The cage size was 60 cm × 60 cm × 40 cm with a temperature of 23–25 °C and a relative humidity of 55%.

### Gross observations of animal statuses and meniscus

Animal body weight was measured, and wound conditions as well as gait were observed and recorded daily. For joint function scores, the knees’ behavioral scores of the three groups of animals were scored according to the absence of passive movement of the knee, the degree of lameness, and the stability of the anterior drawer test, with a minimum score of 0 and a maximum score of 9 at 2nd, 4th, and 8th weeks after surgery with a reference to Du et al. [11]. Six rabbits were randomly sacrificed in the experimental group at the 2nd, 4th, and 8th weeks after the operation. And 2 rabbits in the controls were performed as the experimental group. The knee was experimentally dissected, and the typical meniscus shapes were observed along with other properties including fractures, color, and surface smoothness.

### Histopathological and immunohistochemistry evaluation

The posterior angles of the medial meniscus in the three groups were cut separately, and the surrounding tissue was thoroughly removed. 0.9% sodium chloride brine was rinsed and fixed for 24 h. The histological section of the operational side of the meniscus was stained with hematoxylin-eosin. Histological changes in the meniscus tissue were measured according to the method as previously described [11].

Immunohistochemical staining using antibodies directly against MMP-13 was adopted with the Histostain-SP kit (Zymed, San Francisco, CA). The tissue sections were incubated with a specific antibody against MMP-13 (Santa Cruz Biotechnology, Inc., Santa Cruz, CA) (1:500). Normal goat IgG (1:300) was used as a negative control. The expression of MMP-13 was detected by a biotin-streptavidin-peroxidase system using diaminobenzidine as a chromogen. Counterstaining was carried out with hematoxylin.

### Cytological measurement of knee joint fluid samples

Primary outcomes included levels of IL-1β and IL-17 in the knee joint fluid. For each operative animal, a minimum of 1 ml of synovial fluid was aspirated from the knee joint prior to orthopedic surgery via direct needle aspiration. Samples were placed in a BD Vacutainer test tube and centrifuged at 3000 rpm for 10 min. The supernatant was stored at 20 °C until the assay. The levels of IL-1β and IL-17 were measured by means of an enzyme-linked immunosorbant assay (ELISA) with the Quantikine® HS Human IL-1β and IL-17 Immunoassay kit (R&D Systems, MN, USA). The limit of detection was lower than 2 pg/mL for IL-1β and IL-17, and the intra-batch CV and inter-batch CV were < 9% and < 15% respectively.

### RNA isolation and analysis of the mRNA expressions of MMP-13

0.1 g of fresh meniscus tissue was cut into a foam with scissors. The pellets were collected, and total RNA was extracted with TRIzol reagent (Invitrogen, Carlsbad, CA, USA). cDNA was synthesized with a cDNA synthesis kit (Bio-Rad, Hercules, CA, USA) according to the manufacturer’s instructions. The primer sequences are as follows: MMP-13 forward TGACCACTCCAAGGACCCAG; reverse GAGGATGCAGACGCCCAGAAGA. G3PDH forward CCACTTTGTGAAGCTCATTTCCT; reverse TCGTCCTCCTCTGGTGCTCT.

qRT-PCR analysis of the mRNA was performed using a standard kit (Invitrogen, Carlsbad, CA, USA). The relative transcript levels of the target genes were normalized to that of G3PDH using the 2^−∆∆Ct^ assay. Relative levels of gene expression are presented as the mean ± standard deviation of three independent experiments.

### Statistical analysis

Statistical analysis was performed with SPSS version 10.0 (SPSS, Inc., Chicago, IL, USA), and data are expressed as the mean ± standard deviation. Normality of data distribution was assessed by the Jarque-Bera test. When the cytokine concentrations were non-Gaussian distribution, both *t* test and Mann–Whitney–Wilcoxon (MWW) tests were utilized. Analysis of variance (one-way ANOVA) and post hoc Bonferroni multiple comparisons test was utilized to compare differences between groups. A value of *P* < 0.05 was considered statistically significant.

## Results

### Characteristics of animal statues and meniscus in the ALB and PLB groups

At 2 weeks post-surgery, the incisions in all groups had recovered without any signs of infection or delayed healing and the control group remained healthy. Operated limbs could be gradually moved, and no abnormalities were found. There was no significant difference in the functional score of the operated knee joint of rabbits in the control group at 2 weeks (7.42 ± 0.24), 4 weeks (7.53 ± 0.25), and 8 weeks (7.44 ± 0.25) after surgery (*P* > 0. 05). In the ALB and PLB groups, functional score of the operated knee joint of rabbits at 4 weeks (ALB 5.24 ± 0.24; PLB 5.24 ± 0.24) and 8 weeks (ALB 3.23 ± 0.21; PLB 3.25 ± 0.22) was lower than that of rabbits at 2 weeks (ALB 6.42 ± 0.23; PLB 6.32 ± 0.24) after operation (*P* < 0. 05), and functional score of the operated knee at 8 weeks was lower than that of 4 weeks (*P* < 0. 05). Compare with the same time point, functional scores of the operated knee in the both model groups were significantly lower than those in the control group (*P* < 0. 05). Besides, there was no significant difference in knee joint function score between the two groups at time points (*P* > 0.05).

In the control groups, the structure of the meniscus was complete and the surface was bright white and very smooth and showed no tears each week. In AMB group after 2 weeks, the meniscus was smooth and dark yellow in color, had complete general structure, and showed good toughness with no obvious tears on the body and the free margin. In week 4, the general meniscus structure was still complete. The surface was rough and pale yellow with relaxation and no tears. In week 8, the free margin on the meniscus appeared to be worn out, had a rough surface, was dark yellow, and had poor toughness, and the degree of tension significantly decreased. In the PLB group, the meniscus had an intact structure and poor surface flatness, was pale yellow, and had no tears at the 2nd week. At the 4th week, the inner edge of the meniscus showed tear, and the surface was rough and yellowish, with obvious signs of relaxation. The meniscus body was damaged, the surface flatness had significantly deteriorated, it was deep yellow, the toughness had noticeably deteriorated, and the degree of tension was significantly reduced after 8 weeks.

### Pathological morphology of meniscus on the ALB and PLB groups

**Fig. 1:**
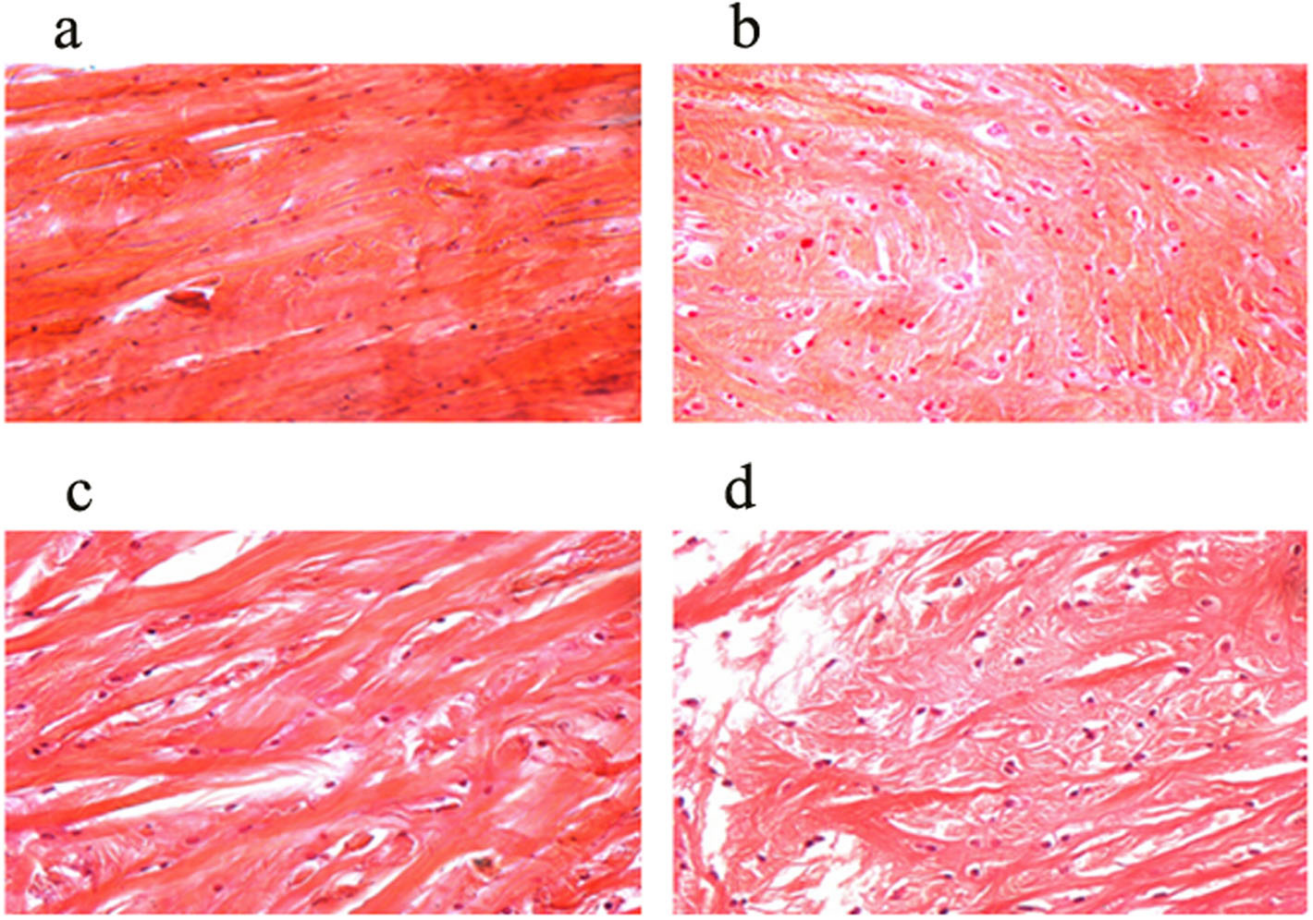
Morphologies of the knee meniscus and HE staining on the rabbits at the 2nd, 4th, and 8th weeks after the operation in the AMB groups and control groups (HE staining ×400). **a** Structures of the knee meniscus and HE staining on the rabbits sacrificed after 2 weeks were imaged in the sham control groups. **b** Structures of the knee meniscus and HE staining on the rabbits sacrificed after 2 weeks were imaged in the AMB groups. **c** Structures of the knee meniscus and HE staining on the rabbits sacrificed after 4 weeks were imaged in the AMB groups. **d** Structures of the knee meniscus and HE staining on the rabbits sacrificed after 8 weeks were imaged in the AMB groups. AMB, anteromedial bundle

**Fig. 2:**
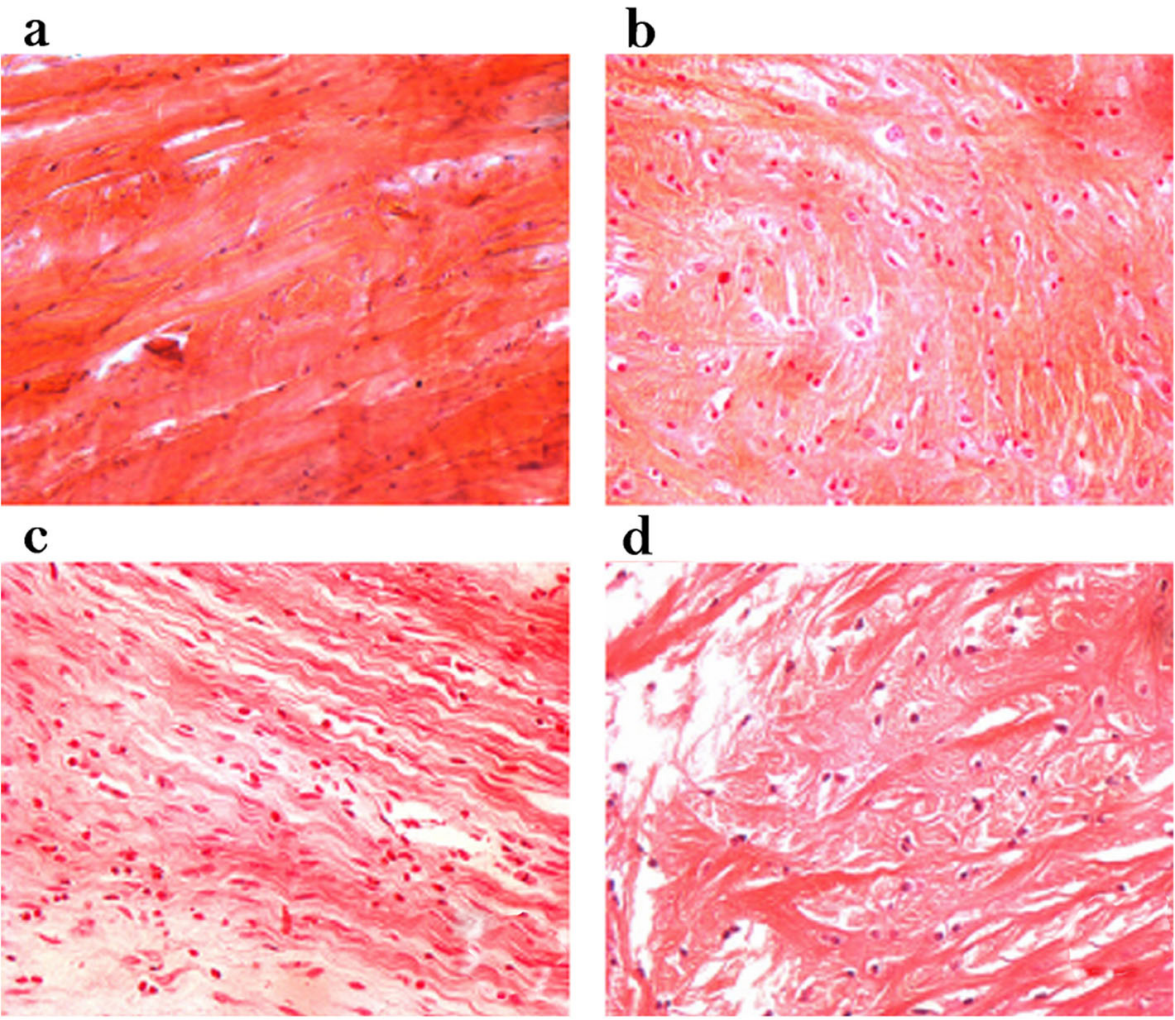
Morphologies of the knee meniscus and HE staining on the rabbits at the 2nd, 4th, and 8th weeks after the operation in the PLB groups and sham control groups (HE staining, × 400). **a** Structures of the knee meniscus and HE staining on the rabbits sacrificed after 2 weeks were imaged in the sham control groups. **b** Structures of the knee meniscus and HE staining on the rabbits sacrificed after 2 weeks were imaged in the PLB groups. **c** Structures of the knee meniscus and HE staining on the rabbits sacrificed after 4 weeks were imaged in the PLB groups. **d** Structures of the knee meniscus and HE staining on the rabbits sacrificed after 8 weeks were imaged in the PLB groups. PLB, posterolateral bundle

In the control groups, the meniscus HE staining characteristics showed that the surface was dense and smooth. Chondrocyte cells and collagen fibers were arranged neatly and were well defined. Non-clustered chondrocytes and non-angiogenesis in addition to inflammatory cells were found (Fig. 1a). In the AMB groups, staining showed that the meniscus surface structure was consistent and complete. The collagen fibers were arranged in a compact area, and only the chondrocyte cells were enlarged (Fig. 1b) for the 2nd week. The staining depth changed, and the surface was uneven. Also, the tissue was loose, and the chondrocyte cells were irregularly arranged (Fig. 1c) at the 4th week. At the 8th week, the meniscus smoothness was significantly reduced, showing that the local slag formation and collagen fiber tissue sequence had altered dramatically and a small amount of inflammatory cells infiltration was observed (Fig. 1d). In addition, the meniscus HE staining exhibited the same characteristics in PLB groups as the AMB groups (Fig. 2a). After 2 weeks, the meniscus was structurally intact, the surface was poorly formed, it was pale yellow, and the collagen fiber was worse and exhibited relaxation. The surface had poor flatness, and the tissue was loose and showed irregular arrangement of chondrocyte cells (Fig. 2b). After 4 weeks, the flatness of the meniscus was weak, and local slag formation was observed. The sequence of the collagen tissue had changed obviously, and a small amount of inflammatory cells had infiltrated into the meniscus (Fig. 2c). After 8 weeks, the smoothness of the meniscus was worse, the tissue was loose, the sequence of the collagen tissue was altered, and the thickness was uneven. A large number of inflammatory cells had also infiltrated into the meniscus (Fig. 2d).

### Expressions of inflammatory factors in knee joint fluid of different groups

Both IL-1β and IL-17 in the synovial fluid showed a demonstrable increase treads from the 2nd to the 8th weeks in the ALB and PLB groups. Compared with the control group, there was a difference between ALB and PLB groups at three time phases (*P* < 0.05) as showed in Tables 1 and 2.

**Table 1:** Comparison of MMP-13, IL-1β, and IL-17 on AMB between the control and experimental groups (*x̄* ± *s*, *n* = 8)

Weeks	Experimental group			Control group			*P* value
	MMP-13 (%)	IL-1β (ng/ml)	IL-17 (ng/ml)	MMP-13 (%)	IL-1β (ng/ml)	IL-17 (ng/ml)	
2	1.56 ± 0.21	17.54 ± 4.60	20.31 ± 8.45	1.28 ± 0.17	1.52 ± 1.60	1.31 ± 0.55	< 0.05*
4	12.24 ± 0.13	21.44 ± 6.70	24.41 ± 7.35	1.47 ± 0.21	1.54 ± 1.70	1.33 ± 0.45	< 0.05*
8	10.53 ± 0.25	25.53 ± 6.30	38.31 ± 6.53	1.17 ± 0.16	1.53 ± 1.50	1.32 ± 0.55	< 0.05*

Data are presented as mean ± standard deviation of three independent experiments, each in triplicates

*AMB* anteromedial bundle, *MMP-13* matrix metalloproteinase-13

*P < 0.05 compared with the sham control group determined by ANOVA and post hoc Bonferroni multiple comparisons test

**Table 2:** Comparison of MMP-13, IL-1β, and IL-17 on PLB between the control and experimental groups (*x̄* ± *s*, *n* = 8)

Weeks	Experimental group			Control group			*P* value
	MMP-13 (%)	IL-1β (ng/ml)	IL-17 (ng/ml)	MMP-13 (%)	IL-1β (ng/ml)	IL-17 (ng/ml)	
2	1.76 ± 0.17	17.54 ± 4.60	20.31 ± 8.45	1.15 ± 0.20	1.52 ± 1.60	1.31 ± 0.55	< 0.05*
4	12.46 ± 0.23	21.44 ± 6.70	24.41 ± 7.35	1.26 ± 0.16	1.54 ± 1.70	1.33 ± 0.45	< 0.05**
8	10.79 ± 0.21	25.53 ± 6.30	38.31 ± 6.53	1.13 ± 0.25	1.53 ± 1.50	1.32 ± 0.55	< 0.05**

Data are presented as mean ± standard deviation of three independent experiments, each in triplicates

*PLB* posterolateral bundle, *MMP-13* matrix metallo proteinase-13

*Significant = *P* < 0.05

**P < 0.05 compared with the sham control group determined by ANOVA and post hoc Bonferroni multiple comparisons test

### Levels of MMP-13 and MMP-13 mRNA in the meniscus of different groups

Immunohistochemical staining for MMP-13 showed the same characteristics in the experimental groups. MMP-13 showed weak positive expression, and both the cytoplasm and matrix were not expressed in the control groups. The surface, matrix, and cytoplasm showed weak positive stain, and the cells were rounded and full and exhibited some cytoplasmic expressions for the 2nd week. The expressions of the surface and interstitial inside the specimen showed strong positive staining, partial degradation of the matrix, relaxation of collagen fibrous tissue, and change in cell morphology at the 4th week. Staining degree of meniscus decreased from 8 weeks; especially cytoplasm, matrix degradation, partial collagen fiber fracture, cell size, and shape were different.

MMP-13-positive cells were counted in 100 images and compared in the three groups. The positive rate of MMP-13 expression in the AMB and PLB groups showed an increase treads from the 2nd to the 4th weeks. Compared with the control group, a significant difference was observed among 2, 4, and 8 weeks (*P* < 0.05). However, the positive rate of MMP-13 expression in the AMB and PLB groups showed a decrease after 8 weeks (Tables 1 and 2).

**Fig. 3:**
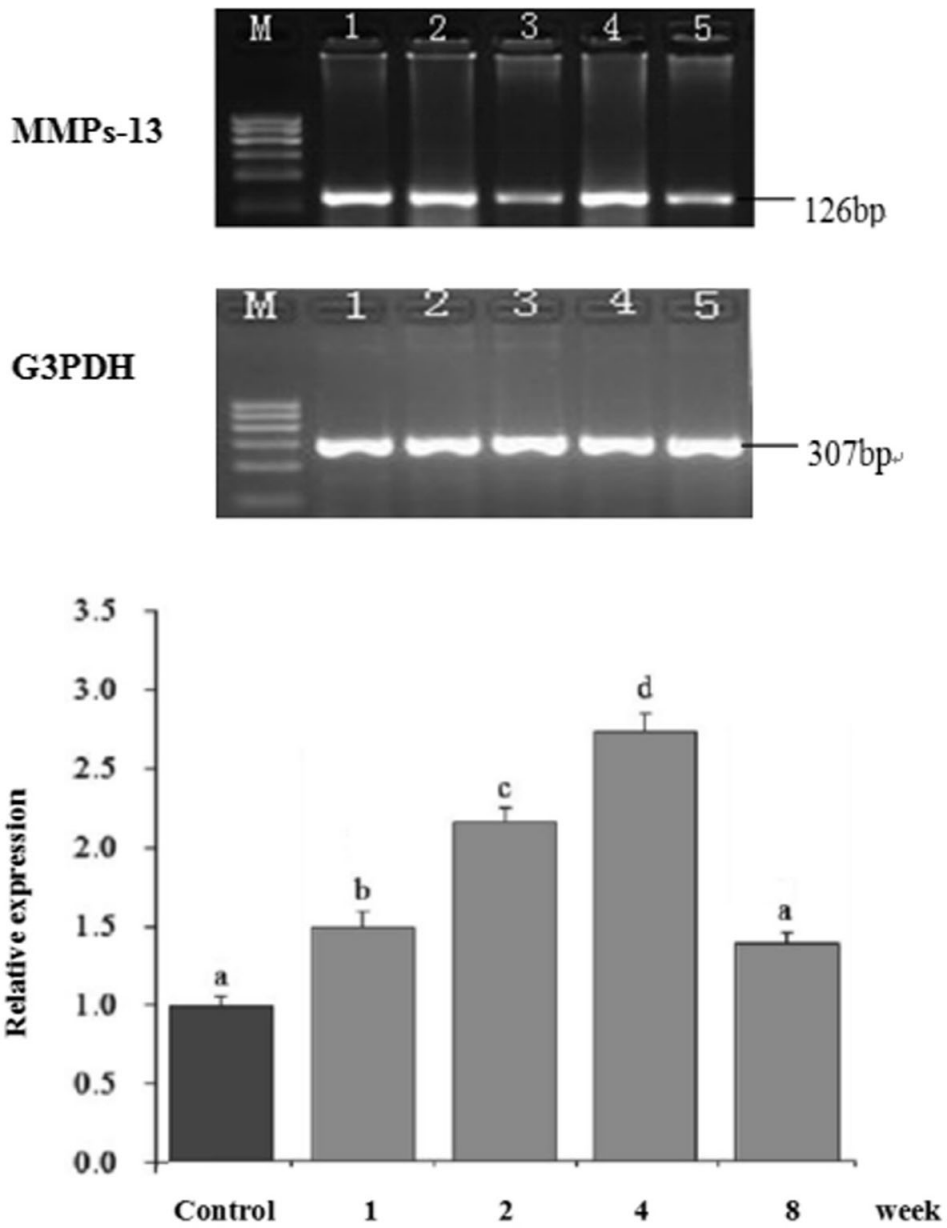
Levels of MMP-13 mRNA in the meniscus of different experimental groups. The expression of MMP-13 mRNA increased from the 2nd week to the peak on the 4th week which was significantly different (*P* < 0.05) compared with the sham control group on the AMB group and the PLB groups. Whilst the level of MMP-13 mRNA decreased at the 8th week in both groups, the difference was not statistically significant (*P* > 0.05) compared with the sham control group. AMB, anteromedial bundle; MMP-13, matrix metalloproteinase-13; M, marker

The expressions of MMP-13 mRNA in the meniscus of the three groups were identified by RT-PCR. The findings demonstrated that the expression of MMP-13 mRNA increased from the 2nd week to peak at the 4th week and the difference was significant (*P* < 0.05) compared to the control group on the AMB group and the PLB group. The level of MMP-13 mRNA decreased in the 8th week in both groups, although a significant difference was found (*P* > 0.05) compared to the control group (Fig. 3).

## Discussion

The ACL is an important stable structure in the knee joint. Its principal role is tantamount to limit the tibial advance and to adjust the stability of the knee joint rotation function [11]. However, the ACL is also the most easily damaged structure in the knee joint [2]. Anatomically, the ACL can be subdivided into the AMB and the PMB [12]. Each function bundle affects the tibial tuberosity advancement and rotation in different tension modes over the entire flexion and extension of the knee joint. If any of these band, it affects the stress distribution for all parts of the knee and can lead to damaging [13, 14]. As the anatomical structure and histochemistry of rabbit knee joints are similar to humans, rabbits were served as experimental models. Knee modeling was utilized to study the pathological process, histopathological features, and cartilage biochemical metabolism of articular cartilage after partial ACL injury [15, 16].

In this study, we successfully established a partial ACL injury model in rabbit knee joint and systematically observed a serial of inflammatory effects on the meniscus at the 2nd, 4th and 8th weeks. The complete healthy meniscus showed bright, white, and smooth. When ACL injury on the discrete bundles occurred, the part of meniscus exhibited gradually unevenness and loose, whose surface become rough and yellow; afterwards, the deteriorated meniscus become relaxed. In a viewpoint of HE staining findings, the injuries of different functional bundles of the ACL were clearly identified as cause of damage to the meniscus. More severe meniscus damages were associated with prolongation of the ACL injury. Immunohistochemistry results showed that MMP-13 was involved in the pathogenesis of meniscus degeneration. Due to the massive damage and death of chondrocytes according to the results of the pathological morphology of meniscus, inflammatory cells increased over time in this study, and the amount of MMP-13 decreased accordingly. It is worth noting that the low expression of MMP13 did not necessarily represent the end of injury and chondrocyte apoptosis.

The meniscus lies in the articular space of the knee joint and also is a key structure of the knee joint. The meniscus acts transfer load and absorbs shock to maintain joint stability which results from vital functions of collagen which is composed of fibrous tissue [17]. The medial collateral ligament is contacted to the medial meniscus, and the fiber is connected with the diaphragm muscle [18]. The meniscus maintains stability and flexibility of the normal physiological function. Some studies have revealed that the knee joint can change the mechanical properties of the medial meniscus after injury of the knee at different angles [19]. When the meniscus angle is considerably higher than the anterior horn, the knee joint tomography suggests that the meniscus of the posterior horn is vulnerable to damage. This can lead to secondary knee medial meniscus damage which requires ACL reconstruction as soon as possible [20].

In the course of knee OA after ACL injury, inflammatory mediators play an important role at the beginning of the process and mechanical factors accelerate progression of the OA [21]. The pathogenesis of ACL injury leading to OA has not been fully elucidated. However, the rotational changes occurring after ACL injury may be a factor in the onset of knee OA in healthy cartilage as a result of the shift of load transfer to the region that does not have frequently load bearing at the tibial-femoral contact of the articular surface during walking. Then, the shift in the load bearing area cause cartilage damage and increase fibrillation of collagen networks and matrix consolidation because of loss of proteoglycans at the surface layer. Eventually, as the cartilage begins to degrade, the disruption of the joint progresses more rapidly with increasing load. The degraded cartilage will produce upregulation of catabolic factors such as MMP and ILs. This finding was in an agreement with the publication of Andriacchi et al. [22]. Studying the mechanism can help physicians choose appropriate treatments for patients including surgery, drugs, and tissue engineering to reduce the incidence of OA [23].

In the present study, we measured the levels of IL-1β and IL-17 in synovial fluid concerning the role of inflammatory mediators in the process of chronic degeneration in the knee meniscus during 8 weeks. Our findings indicated that the elevated levels of IL-1β and IL-17 suggested that inflammatory factors were involved in the degeneration of the meniscus. In previous studies, several studies have confirmed that IL-1β and IL-17 within synovial inflammation were associated with meniscus or cartilage degeneration as primary inflammatory mediators in humans or animals. Our study was in accord with these reporters [24, 25]. High levels of IL-1β and IL-17 in knee traumatic areas demonstrated the level of meniscus or cartilage damage and the presence of a local inflammatory response that triggers early knee OA [26]. IL-17 is also a pro-inflammatory cytokine, which has been declared to increase synovitis and joint destruction following intra-articular injection. IL-17 is mainly secreted by Th17 and can promote the proliferation and activation of T cells. Activated T cells develop a large number of cytokines such as IL-1β to accelerate meniscus injury. IL-1β can inhibit the proliferation of chondrocytes by interfering with the metabolism of meniscus chondrocytes and activate the MMPs signaling pathway. Studies indicated that a variety of inflammatory mediators play critical roles in the pathogenesis of knee trauma and OA progression. Expressions of various matrix MMPs are significantly increased, including MMP-1, MMP-2, MMP-3, and MMP-7 in addition to MMP-13 [27]. The increase in MMPs triggers a series of biological reactions that increase collagen degradation in the cartilage matrix and destroy meniscus integrity.

Current surgical treatment of the part ACL injury is different from bundle injury diagnosis and remains controversial [28, 29]. The ACL part injury is a common ACL injury model; however, the treatment standards of patients with ACL injury are inconsistent [30, 31]. An orthopedic expert reported that patients with ACL part injury, which had been followed up for 9 to 15 years, have developed a complete rupture, but only 32% of patients recovered to pre-injury levels. Forty-eight percent of patients had a poor prognosis, and 86% of patients had persistent symptoms [32]. The complete breakdown of the ACL single bundle injury may depend on the number of damaged fibers, the type of injury, and the presence of secondary injury [33, 34]. These factors may be particularly important after PLB injury and impact the stability of the knee joint [35, 36]. According to the results of the study, the meniscus degradation began earlier in PLB groups compared to ALB groups.

There are some limitations to our study. First, the models were assessed at the 2nd, 4th, and 8th weeks; there remained a lack of long-term follow-up observation till 12 weeks or more long time. Second, there was the absence of ACL reconstruction model to observe the effect on the meniscus. In addition, the study did not include the histology quantification of any grading scale to evaluate the macroscopic appearance of the tissues. All these considerations will be validated in the future research.

## Conclusions

We successfully established models with distinctive damage to functional bundle on ACL in the rabbit knee. Our findings illustrated that the elevated levels of IL-1β and IL-17 increased MMP13 expression in the posterior horn of the medial meniscus in the rabbit knee joint model with partial ACL injury, resulting in meniscus degradation and knee OA occurrence.

## Data Availability

All data generated or analyzed during this study are available in this published article.

## Abbreviations

ACL: Anterior cruciate ligament
AMB: Anteromedial bundle
PLB: Posterolateral bundle
IL-1β: Interleukin-1β
IL-17: Interleukin-17
OA: Osteoarthritis
MMP: Matrix metalloproteinase
ELISA: Enzyme-linked immunosorbant assay
CV: Coefficient of variation
